# How Well Have Projected Lung Cancer Rates Predicted the Actual Observed Rates?

**DOI:** 10.31557/APJCP.2021.22.2.437

**Published:** 2021-02

**Authors:** Qingwei Luo, Julia Steinberg, Xue Qin Yu, Michael Caruana, Karen Canfell, Dianne L O’Connell

**Affiliations:** 1 *Cancer Research Division, Cancer Council NSW, Sydney, Australia. *; 2 *The University of Sydney School of Public Health, Faculty of Medicine and Health, the University of Sydney, Sydney, Australia. *; 3 *Prince of Wales Clinical School, University of New South Wales, Sydney, Australia. *; 4 *School of Medicine and Public Health, University of Newcastle, Newcastle, Australia. *

**Keywords:** Statistical projections, incidence rates, mortality rates, age-period, cohort model, generalized linear model

## Abstract

**Background:**

While many past studies have constructed projections of future lung cancer rates, little is known about their consistency with the corresponding observed data for the time period covered by the projections. The aim of this study was to assess the agreement between previously published lung cancer incidence and/or mortality rate projections and observed rates.

**Methods:**

Published studies were included in the current study if they projected future lung cancer rates for at least 10 years beyond the period for which rates were used to obtain the projections, and if more recent observed rates for comparison covered a minimum of 10 years from the beginning of the projection period. Projected lung cancer incidence and/or mortality rates from these included studies were extracted from the publications. Observed rates were obtained from cancer registries or the World Health Organization’s Mortality Database. Agreement between projected and observed rates was assessed and the relative difference (RD) for each projected rate was calculated as the percentage difference between the projected and observed rates.

**Results:**

A total of 59 projections reported in 14 studies were included. Nine studies provided projections for 20 years or more. RDs were higher for those projections in which the lung cancer rates peaked during the projection period, and RDs increased substantially with the length of the projection period. When lung cancer rates peaked during the projection period, methods incorporating smoking data were generally more successful at predicting the trend reversal than those which did not incorporate smoking data. Mean RDs for 15-year projections comparing methods with or without smoking data were 12.7% versus 48.0% for males and 8.2% versus 42.3% for females.

**Conclusions:**

The agreement between projected and observed lung cancer rates is dependent on the trends in the observed rates and characteristics of the population, particularly trends in smoking.

## Introduction

Lung cancer has been the most commonly diagnosed cancer in the world for several decades, and is the leading cause of cancer death worldwide (Ferlay et al., 2018). Reliable projections of future patterns of lung cancer incidence and mortality are therefore very important for health service planning (Bashir and Esteve, 2001). Projecting future trends in cancer incidence and mortality is always complicated, as the population’s risk factor profile will change over time, and in some cases there is a significant latency period between risk factor exposure and cancer development (Bray and Moller, 2006). For lung cancer in particular, the well-documented association between tobacco smoking and cancer risk means that the accuracy of any projections is very reliant on how smoking behaviours are accounted for in the projection methods (Brown and Kessler, 1988; Shibuya et al., 2005; Luo et al., 2018). As detailed data on smoking behaviours are not always available, the selection and implementation of an appropriate projection method is complex. 

A systematic review identified 101 studies published between 1st January 1988 and 14th August 2018 that used statistical methods to project lung cancer incidence or mortality rates (Yu et al., 2019). The aims of this study were to compare previously published lung cancer incidence and/or mortality rate projections to observed data that became available since their publication, and to provide insights into key factors that should be considered when selecting methods for projecting lung cancer rates. 

## Materials and Methods

*Selection criteria*

**Figure 1:**
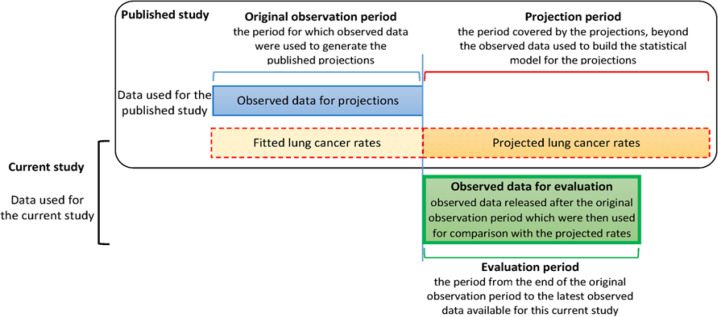
Illustration of the Time Periods Covered in the Published Studies and the Evaluation Period for the Current Study

The literature search (Online resource 1) and review protocol for potentially relevant studies are described in detail in our previously published systematic review (Yu et al., 2019), and the full inclusion and exclusion criteria are summarised in Online resource 2. The results of the literature search and the process for selecting studies are described in Online resource 3. Published studies were included in the current study if they projected future lung cancer rates for at least 10 years beyond the period for which rates were used to obtain the projections, and if more recent observed rates for comparison covered a minimum of 10 years from the beginning of the projection period. We defined the ‘original observation period’, ‘projection period’, ‘evaluation period’ and ‘observed data for evaluation’ as follows (illustrated in Figure 1). For each study, the ‘original observation period’ is the period for which observed data were used to generate the published projections. The ‘projection period’ is the period covered by the projections, beyond the observed data used to build the statistical model. ‘Observed data for evaluation’ are the more recently released observed rates which could be compared with the projected rates. The ‘evaluation period’ is the period from the beginning of the projection period to the latest observed data available for this current study.

*Data extraction*

Individual projections were the unit of analysis in this study so that publications that used more than one projection method or multiple datasets for different countries contributed multiple projections. Predicted lung cancer incidence/mortality rates from the published studies, including the fitted values for the data period used for model fitting and the projected values for the period beyond the original observed data, were extracted from the publications (Online resource 4). Newly released observed data for evaluation were obtained from the World Health Organization (WHO) Mortality Database (World Health Organization, 2017), United States Cancer Statistics (SEER, 2016), NORDCAN (Engholm et al., 2017), Cancer Statistics Registrations England at the National Archives (Office for National Statistics, 2016), and the Bulgarian National Cancer Registry (Bulgarian National Cancer Registry, 2018). These observed data were age-standardised to the same standard population used in the published studies, including the WHO World standard population (Ahmad et al., 2001), Segi World standard population, European standard population, the 1970 and 2000 USA standard populations (SEER, 2018), and the 1985 Japanese standard population (Kuroishi et al., 1992). In order to summarise the differences and similarities between the methods used for projections, we applied our previously developed organisational framework to group these methods (Yu et al., 2019).

*Statistical analyses*

The aim of this evaluation was not to test the exact agreement between the projected and observed rates. Indeed, a formal test was often not possible as it requires estimates of standard errors for the projected rates, which were not always available. Instead, two measures were used to evaluate the overall performance of each projection: assessment of the agreement in the overall trends, and the relative difference (RD) of the projected age-standardised rate (ASR) compared to the observed ASR.

The graphed projected and observed cancer rates were visually inspected to assess the agreement in the overall trends, and in particular whether the projections predicted the peak in the lung cancer rates. The peak in the cancer rate was defined as the point at which there was a significant change in the lung cancer rates from an increasing trend to a decreasing trend, as identified by the Joinpoint regression program with p<0.05 considered to denote statistical significance (Kim et al., 2000). As most of the studies used 5-year grouped data, we determined that ‘lung cancer rates peaked in the original observation period’ if the significant change point identified by Joinpoint regression occurred at least 5 years before the end of the original observation period.

The second measure (RD) compared the projected ASR to the observed data, and was defined as:

$\mathrm{RD}=100\times \frac{\left|E_{t}-O_{t}\right|}{O_{t}}$ *, t=1,2,…, n*

where E_t_ is the projected ASR and O_t_ is the observed ASR, and t is the year of the projection beyond the original observation period. RD was calculated for each year for which projections were available for evaluation, and at the 10-year, 15-year and 20-year points in the projection period where available. The mean RD for a set of projections was calculated as the mean of the RDs for the projections in this set. The RD can be interpreted as a measure of the closeness of the observed and projected values.

**Figure 2:**
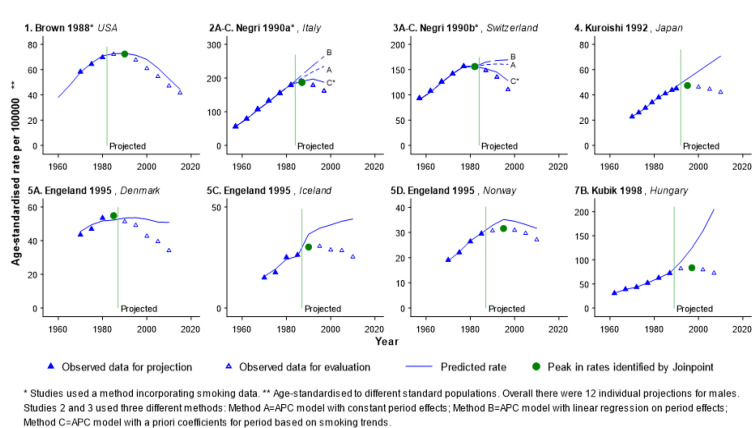
Projections in which a Statistically Significant Change in Lung Cancer Rates Occurred during theProjection Period, Males

**Figure 3:**
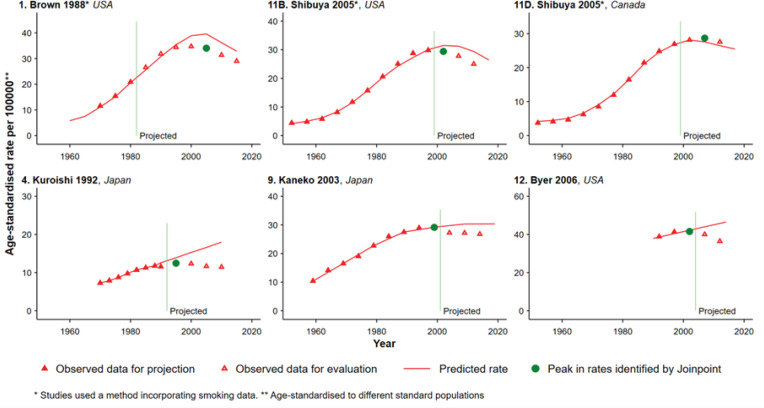
Projections in which a Statistically Significant Change in Lung Cancer Rates Occurred during the Projection Period, Females

**Figure 4:**
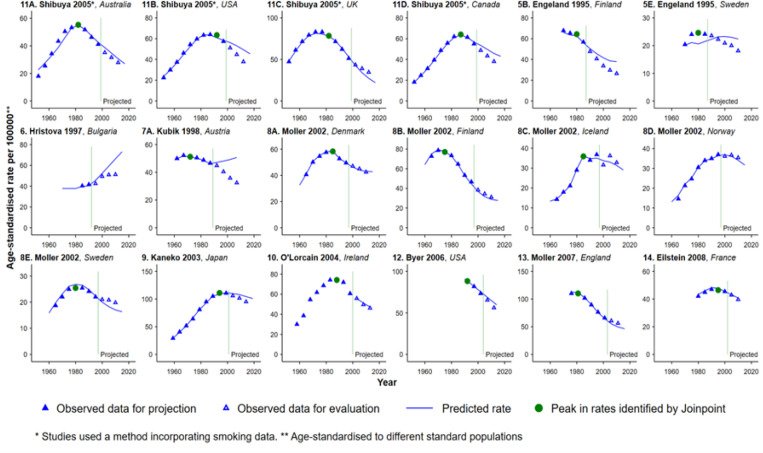
Projections in which a Statistically Significant Change in Lung Cancer Rates did not Occur during the Projection Period, Males

**Table 1:** Relative Differences in Projected Lung Cancer Rates for Males

First author and publication year		Country	Statistical projection method used	Relative difference (%)			
				10-year projection	15-year projection	20-year projection	Direction of differences*
All projections for males				13.6	25.9	50.7	
Rate peaked during the projection period				20.4	39.1	59.1	
Methods incorporating smoking				6.2	12.7	11.7	
1	Brown 1988	USA	GLM with smoking variable	0.7	5.8	11.7	Overestimate
2C	Negri 1990a	Italy	APC model with a priori coefficients for period based on smoking trends	10.6	16.2		Overestimate
3C	Negri 1990b	Switzerland	APC model with a priori coefficients for period based on smoking trends	7.3	16.1		Overestimate
Methods not incorporating smoking				25.1	48	68.5	
2A	Negri 1990a	Italy	APC model with constant period effects	22.9	45.2		Overestimate
2B	Negri 1990a	Italy	APC model with linear regression on period effects	33.2	63.4		Overestimate
3A	Negri 1990b	Switzerland	APC model with constant period effects	19.3	45.1		Overestimate
3B	Negri 1990b	Switzerland	APC model with linear regression on period effects	24.5	53.2		Overestimate
4	Kuroishi 1992	Japan	Other GLMs	27.0	45.4	67.8	Overestimate
5A	Engeland 1995	Denmark	Other GLMs	9.1	23.6	29.3	Overestimate
5C	Engeland 1995	Iceland	Other GLMs	29.5	43.1	51.1	Overestimate
5D	Engeland 1995	Norway	Other GLMs	11.3	11.5	11.8	Overestimate
7B	Kubik 1998	Hungary	APC model	49.1	101.3	182.7	Overestimate
Rate did not peak during the projection period				9.2	14.5	33.9	
Rate peaked during the observation period				9.8	15.3	33.9	
Methods incorporating smoking				13.9	20.5		
11A	Shibuya 2005	Australia	GLM with smoking variable	9.9	10.1		Overestimate
11B	Shibuya 2005	USA	GLM with smoking variable	18.6	30.6		Overestimate
11C	Shibuya 2005	UK	GLM with smoking variable	12.6	21.1		Underestimate
11D	Shibuya 2005	Canada	GLM with smoking variable	14.4	20.2		Overestimate
Methods not incorporating smoking				8.5	12.7	33.9	
5B	Engeland 1995	Finland	Other GLMs	11.8	23	30.2	Overestimate
5E	Engeland 1995	Sweden	Other GLMs	4.1	11.4	14.9	Overestimate
7A	Kubik 1998	Austria	APC model	18.9	37.4	56.5	Overestimate
8A	Moller 2002	Denmark	APC model	2.1	1.3		Underestimate
8B	Moller 2002	Finland	APC model	8.4	7.3		Underestimate
8C	Moller 2002	Iceland	APC model	8.8	4.4		Underestimate
8E	Moller 2002	Sweden	APC model	12.2	14.1		Underestimate
9	Kaneko 2003	Japan	APC model	6.4			Overestimate
10	O'Lorcain 2004	Ireland	Other GLMs	3.2	2.3		Overestimate
12	Byers 2006	USA	Other GLMs	10.9			Overestimate
13	Moller 2007	England	APC model	11.9			Underestimate
14	Eilstein 2008	France	APC model	2.9			Overestimate
Rate did not peak during both the observation and projection periods				3.8	10		
6	Hristova 1997	Bulgaria	APC model	5.9	16.4		Overestimate
8D	Moller 2002	Norway	APC model	1.7	3.5		Underestimate

* For most projections RDs at the 10-year, 15-year and 20-year points are in the same direction, with the exception of 5E with three of the four being overestimates and 8C with two of the three being underestimates. APC, age-period-cohort; GLM, generalised linear model; RD, relative difference; UK, United Kingdom; USA, United States of America.

**Figure 5:**
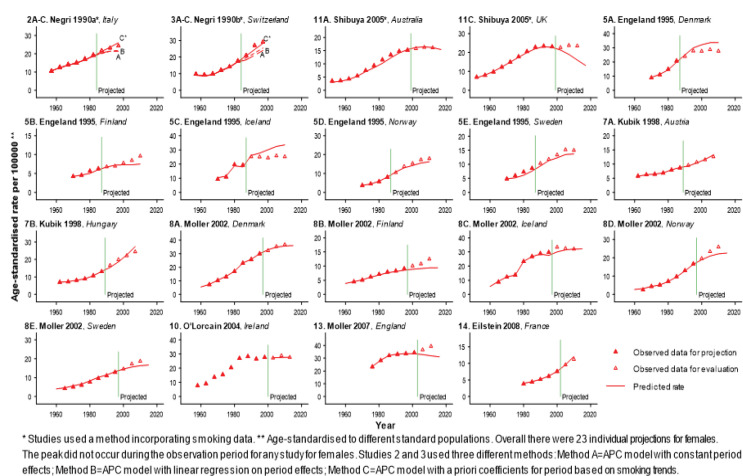
Projections in which a Statistically Significant Change in Lung Cancer Rates did not Occur during the Projection Period, Females

**Table 2:** Relative Differences in Projected Lung Cancer Rates for Females

First author and publication year		Country	Statistical projection method used	Relative difference (%)			Direction of differences
				10-year projection	15-year projection	20-year projection	
All projections for females				9.6	11.3	18.3	
Rate peaked during the projection period				13.4	16.7	34.5	
Methods incorporating smoking				6.5	8.2	12	
1	Brown 1988	USA	GLM with smoking variable	3.2	2.9	12	Overestimate
11B	Shibuya 2005	USA	GLM with smoking variable	12.3	17.6		Overestimate
11D	Shibuya 2005	Canada	GLM with smoking variable	3.9	4		Underestimate
Methods not incorporating smoking				20.4	42.3	57	
4	Kuroishi 1992	Japan	Other GLMs	24	42.3	57	Overestimate
9	Kaneko 2003	Japan	APC model	11.4			Overestimate
12	Byers 2006	USA	Other GLMs	25.7			Overestimate
Rate did not peak during both the observation and projection periods				8.6	10.2	13.6	
Methods incorporating smoking				7.7	10.3		
2C	Negri 1990a	Italy	APC model with a priori coefficients for period based on smoking trends	0.3	6.1		Overestimate
3C	Negri 1990b	Switzerland	APC model with a priori coefficients for period based on smoking trends	9.2	2.9		Underestimate
11A	Shibuya 2005	Australia	GLM with smoking variable	1.2	0.4		Overestimate
11C	Shibuya 2005	UK	GLM with smoking variable	20.1	31.9		Underestimate
Methods not incorporating smoking				8.8	10.2	13.6	
2A	Negri 1990a	Italy	APC model with constant period effects	8.7	11.4		Underestimate
2B	Negri 1990a	Italy	APC model with linear regression on period effects	8.4	12.7		Underestimate
3A	Negri 1990b	Switzerland	APC model with constant period effects	17.1	12.8		Underestimate
3B	Negri 1990b	Switzerland	APC model with linear regression on period effects	21.9	17.7		Underestimate
5A	Engeland 1995	Denmark	Other GLMs	7.8	16.3	17.1	Overestimate
5B	Engeland 1995	Finland	Other GLMs	3.6	5.8	13.7	Underestimate
5C	Engeland 1995	Iceland	Other GLMs	10.5	23.6	25.6	Overestimate
5D	Engeland 1995	Norway	Other GLMs	5.2	7.4	10.4	Underestimate
5E	Engeland 1995	Sweden	Other GLMs	4.2	7.9	11.2	Underestimate
7A	Kubik 1998	Austria	APC model	2.3	0.9	4.9	Overestimate
7B	Kubik 1998	Hungary	APC model	6.6	1.7	12.4	Overestimate
8A	Moller 2002	Denmark	APC model	1.3	2.8		Underestimate
8B	Moller 2002	Finland	APC model	15.3	24.9		Underestimate
8C	Moller 2002	Iceland	APC model	3.1	0.2		Underestimate
8D	Moller 2002	Norway	APC model	10.5	14.4		Underestimate
8E	Moller 2002	Sweden	APC model	9.6	12.7		Underestimate
10	O'Lorcain 2004	Ireland	Other GLMs	4.5	0.3		Underestimate
13	Moller 2007	England	APC model	18.9			Underestimate
14	Eilstein 2008	France	APC model	7.4			Overestimate

APC, age-period-cohort; GLM, generalised linear model; UK, United Kingdom; USA, United States of America.

## Results

A total of 14 eligible studies published between 1988 and 2008 were included, covering 18 countries or regions ranked as very high or high on the Human Development Index (HDI) (Online resource 4) (Brown and Kessler, 1988; Negri et al., 1990a; Negri et al., 1990b; Kuroishi et al., 1992; Engeland et al., 1995; Hristova et al., 1997; Kubik et al., 1998; Moller et al., 2002; Kaneko et al., 2003; O’Lorcain and Comber, 2004; Shibuya et al., 2005; Byers et al., 2006; Moller et al., 2007; Eilstein et al., 2008). Eleven studies reported projections of lung cancer mortality rates and 3 studies reported projections of lung cancer incidence rates. Nine studies provided projections for 20 years or more. Twelve studies used methods that did not incorporate smoking data and 4 studies used methods that did. Two of 14 studies used three methods: age-period-cohort (APC) model with constant period effects, APC model with linear regression on period effects and APC model with a priori coefficients for period effects based on smoking trends (Negri et al., 1990a; Negri et al., 1990b). Three studies reported projections for four or more countries using the same method (Studies 5, 8 and 11). Consequently, there were 30 and 29 individual projections of lung cancer rates for males and females, respectively. 

In general, the RDs were higher for projections in which the lung cancer rates peaked during the projection period, and the RD increased substantially with the length of the projection period (Tables 1 and 2). The RDs for projections for males were higher than those for projections for females. Mean RDs were 13.6%, 25.9% and 50.7% for 10-year, 15-year, and 20-year projections for males (Table 1) and 9.6%, 11.3% and 18.3%, for 10-year, 15-year, and 20-year projections for females (Table 2). In each of the three studies that reported projections for multiple countries using the same method, the RDs for 15-year projections varied between countries, with absolute differences of 12-42% (Studies 5, 8 and 11).

In fewer than one-third of all projections (18 of 59 projections: 12 for males and 6 for females) the lung cancer rates peaked during the projection period (Figures 2 and 3), and nearly all of these projections overestimated the true rate (12 of 12 for males and 5 of 6 for females). All 6 projections which used a method incorporating smoking data (projections 1, 2C and 3C for males in Figure 2; projections 1, 11B and 11D for females in Figure 3), and 1 of 12 projections which did not incorporate smoking data (projection 5D for males in Figure 2) captured the change in the direction of the trend in the lung cancer rate. The RDs for projections which used a method incorporating smoking data (Tables 1 and 2, e.g. mean RDs for 15-year projections were 12.7% and 8.2% for males and females respectively) were consistently lower than those for projections which used methods which did not incorporate smoking data (Tables 1 and 2, e.g. mean RDs for 15-year projections were 48.0% and 42.3% for males and females respectively). This pattern was also demonstrated in the two studies which reported comparisons of methods which did or did not incorporate smoking data using the same lung cancer mortality data (Figures 2 and 5, studies 2A-C and 3A-C).

A statistically significant peak in lung cancer rates did not occur during the projection periods for 41 projections (18 for males and 23 for females), although in 16 projections for males the rates did peak during the original observation period (11A-D, 5B, 5E, 7A, 8A-C, 8E, 9, 10, 12-14 for males in Figure 4), and in some datasets for females the rates appear to have levelled off during the projection period (11A, 11C, 5A, 5C, 10 for females in Figure 5). The peak in lung cancer rates had not occurred during the original observation period for any of the projections for females. For the 41 projections in which the peak in lung cancer rates did not occur during the projection period, most of the studies appear to have good agreement between the projected rates and the more recent observed rates (Figures 4 and 5). The RDs for this set of projections are similar, even when different methods were originally used, and the RDs are consistently lower than for projections where the lung cancer rates peaked during the projection period (Tables 1 and 2). For 15-year projections with a RD>10%, the majority of projections for males were overestimates (7 out of 9; Table 1), while for females 80% of the projections were underestimates (8 out of 10; Table 2).

## Discussion

There have been many studies published which have used various statistical methods to project lung cancer rates, and many of these studies have been frequently referenced in the literature, reflecting the potential high impact and implications for such cancer research. However, very little is known about the consistency between projected and observed lung cancer rates. The statistical methods for projecting lung cancer rates included in this evaluation ranged from simple linear regression to more complex APC models which require specific techniques and software packages. We found that the agreement between published projections and observed actual rates varied by sex and data setting and is largely dependent on whether or not the lung cancer rates peaked during the projection period rather than the original observation period. Our results showed that lung cancer projections for females generally tended to more closely resemble the observed patterns than projections for males. This is likely to be because lung cancer rates for females tended to be more stable throughout the study periods, without the sharp changes that have occurred in the lung cancer rates for males. 

We found that for both males and females, almost all of the projections were overestimates when there was a significant change in the actual lung cancer rates during the projection period, and the RDs were much lower for studies that used a method which incorporated smoking data compared with those that did not do this. Due to the well-established and strong association between smoking exposure and lung cancer risk (Doll and Hill, 1950), the past smoking behaviour in the population should be taken into account when performing lung cancer projections. This is particularly important if sharp changes in smoking trends have occurred (Lopez et al., 1994), since a projection method that does not incorporate the smoking data may not reflect the future impact of these changes in smoking behaviour (Brown and Kessler, 1988). Two of the published studies reported projections of lung cancer mortality rates in Italy and Switzerland by comparing three methods using the same data, and their results confirmed that a method incorporating information on smoking trends more successfully predicted changes in lung cancer mortality rates (studies 2A-C and 3A-C in Figures 2 and 5). However, it should be stressed that the use of a method incorporating smoking data by no means guarantees the reliability of the projection (e.g. see projection 11C in Figure 5), possibly due to variation in the quality of the smoking data, or incomplete capture of other factors that contributed to the changes in lung cancer rates. 

There is no single “best” method for projecting lung cancer rates that suits all situations, as the influences of changing risk factors, diagnostic practice and treatment, are complex and very hard to predict and capture (Cancer Projections Network, 2010). Results from this study show that projections using the same method applied to different study populations still had varying degrees of success in predicting the more recently observed patterns (Moller et al., 2003). For example, a large variation in RDs was observed for studies which reported projections for four or more countries using the same method (Studies 5, 8 and 11). In addition, for populations where the tobacco epidemic was fully established early on and in which the peak in lung cancer rates occurred during the original observation period, or for populations in which the peak in lung cancer rates did not occur at any time during the original observation or projection periods, some methods not incorporating smoking data also provided generally reliable projections for 10-15 years beyond the original observation period (Study 12 in Figure 4). Therefore, our study highlights the importance of selecting appropriate methods for projections based on the observation period, length of the projection period beyond the observed data, data quality and availability, and a good understanding of the tobacco epidemic in the population, as well as any other potential factors that may contribute to changes in lung cancer rates. Moreover, an appropriate validation of the selected projection model should be performed and justified whenever this is possible, as such information is useful for checking the specifications of the model and helps researchers understand its potential limitations.

This study has some limitations. As it is an evaluation of past projections in different data settings over different study periods, some of these studies are not directly comparable to each other. A further potential limitation of this study is that the data for some of the included studies were extracted from figures using computer software. However, to ensure the reliability of this data extraction, it was independently conducted by two authors and the mean values of the two extractions were used for analyses. Also, the agreement between the two extractions was evaluated and found to be high. Finally, it is important to note that this evaluation study is limited to projections of lung cancer incidence or mortality rates only, which are strongly associated with past tobacco exposure. Therefore, the interpretation of the results may not be generalisable to projections of rates for other cancer types.

Despite these limitations this study also has many strengths. It is the first study to provide an objective assessment of previously published projections of lung cancer incidence or mortality rates using newly released observed data. Included studies were identified from a systematic review of statistical methods for projecting lung cancer rates. Furthermore, the measures for evaluation developed in this study provided an objective assessment of the agreement between newly released observed data and the published lung cancer projections. The approach developed in this study may be applicable to evaluations of other disease rate projections.

By comparing newly released cancer statistics with previously reported projected rates for different populations, it is hoped that this study can provide important information for researchers about the applicability and suitability of various methods in different data settings, so that appropriate methods can be chosen to suit the situation and projection requirements for future research.

## References

[B1] Ahmad OB, Boschi-Pinto C, Lopez AD (2001). Age standardization of rates: A new WHO standard [Online]. GPE Discussion Paper Series: No. 31. World Health Organization.

[B2] Bashir SA, Esteve J (2001). Projecting cancer incidence and mortality using Bayesian age-period-cohort models. J Epidemiol Biostat.

[B3] Bray F, Moller B (2006). Predicting the future burden of cancer. Nat Rev Cancer.

[B4] Brown CC, Kessler LG (1988). Projections of lung cancer mortality in the United States: 1985-2025. J Natl Cancer Inst.

[B5] Bulgarian National Cancer Registry (2018). Cancer Incidence in Bulgaria [Online].

[B6] Byers T, Barrera E, Fontham ET (2006). A midpoint assessment of the American Cancer Society challenge goal to halve the U. S. cancer mortality rates between the years.

[B7] Doll R, Hill AB (1950). Smoking and Carcinoma of the Lung. Br Med J.

[B8] Eilstein D, Uhry Z, Lim TA, Bloch J (2008). Lung cancer mortality in France Trend analysis and projection between 1975 and 2012, using a Bayesian age-period-cohort model. Lung Cancer.

[B9] Engeland A, Haldorsen T, Tretli S (1995). Prediction of cancer mortality in the Nordic countries up to the years 2000 and 2010, on the basis of relative survival analysis A collaborative study of the five Nordic cancer registries. APMIS Supplement.

[B12] Hristova L, Dimova I, Iltcheva M (1997). Projected cancer incidence rates in Bulgaria, 1968-2017. Int J Epidemiol.

[B13] Kaneko S, Ishikawa KB, Yoshimi I (2003). Projection of lung cancer mortality in Japan. Cancer Sci.

[B14] Kim HJ, Fay MP, Feuer EJ, Feuer EJ, Midthune DN (2000). Permutation tests for joinpoint regression with applications to cancer rates. Stat Med.

[B15] Kubik A, Plesko I, Reissigova J (1998). Prediction of lung cancer mortality in four Central European countries, 1990-2009. Neoplasma.

[B16] Kuroishi T, Hirose K, Tominaga S, Ogawa H, Tajima K (1992). Prediction of future cancer mortality in Japan. Jpn J Clin Oncol.

[B17] Lopez AD, Collishaw NE, Piha T (1994). A descriptive model of the cigarette epidemic in developed countries. Tob Control.

[B18] Luo Q, Yu XQ, Wade S (2018). Lung cancer mortality in Australia: Projected outcomes to 2040. Lung Cancer.

[B19] Moller B, Fekjaer H, Hakulinen T (2003). Prediction of cancer incidence in the Nordic countries: empirical comparison of different approaches. Stat Med.

[B20] Moller B, Fekjaer H, Hakulinen T (2002). Prediction of cancer incidence in the Nordic countries up to the year 2020. Eur J Cancer Prev.

[B21] Moller H, Fairley L, Coupland V (2007). The future burden of cancer in England: incidence and numbers of new patients in 2020. Br J Cancer.

[B22] Negri E, La Vecchia C, Decarli A, Boyle P (1990a). Projections to the end of the century of mortality from major cancer sites in Italy. Tumori.

[B23] Negri E, La Vecchia C, Levi F (1990b). The application of age, period and cohort models to predict Swiss cancer mortality. J Cancer Res Clin Oncol.

[B24] O’Lorcain P, Comber H (2004). Lung cancer mortality predictions for Ireland 2001-2015 and current trends in North Western Europe. Lung Cancer.

[B25] Office for National Statistics (2016). Cancer Statistics Registrations, England (Series MB1), No.42 [Online].

[B26] SEER (2016). Surveillance, Epidemiology, and End Results (SEER) Program, SEER*Stat Database: Incidence - SEER 9 Regs Research Data, Nov 2017 Sub (1973-2014) [Online].

[B27] SEER (2018). Standard Population Data, Surveillance, Epidemiology, and End Results (SEER) Program [Online].

[B28] Shibuya K, Inoue M, Lopez AD (2005). Statistical modeling and projections of lung cancer mortality in 4 industrialized countries. Int J Cancer.

[B29] World Health Organization (2017). Mortality database [Online].

[B30] Yu XQ, Luo Q, Hughes S (2019). Statistical projection methods for lung cancer incidence and mortality: a systematic review. BMJ Open.

